# Isolated Urethral Injury Without Penile Fracture: A Case Report

**DOI:** 10.7759/cureus.96089

**Published:** 2025-11-04

**Authors:** Tolulope T Ogunfowora, Sadiq Abu, Quadri A Sanni, Andrea Ginepri, Khaled Bseikri

**Affiliations:** 1 Urology, South Warwickshire University NHS Foundation Trust, Warwick, GBR

**Keywords:** flexible cystoscopy, isolated urethral injury, penile fracture, peri-urethral abscess, retrograde urethrogram, scrotal abscess, spongiosal injury, suprapubic cystostomy

## Abstract

An isolated urethral injury without a penile fracture, particularly one resulting from sexual intercourse, is an infrequent occurrence. Urethral injury without concomitant penile fracture poses a diagnostic dilemma, being an uncommon clinical condition. Early recognition and prompt treatment are essential to prevent complications, which sparked this interest. We report a case of isolated proximal bulbar urethral injury without penile fracture following sexual intercourse in an 18-year-old male who presented with two days of peno-scrotal swelling. An initial diagnosis of bilateral epididymoorchitis was made. During admission, a urethral injury was suspected and subsequently confirmed. A peri-urethral abscess and a right hemiscrotal abscess complicated this injury. He was evaluated using an initial ultrasound testes and subsequently a CT scan of the abdomen and pelvis, and a pelvic MRI due to a diagnostic dilemma. He was treated with antibiotics, underwent urinary diversion via suprapubic cystostomy, flexible cystoscopic-guided urethral catheterisation, and scrotal exploration with drainage of peri-urethral and scrotal abscesses. A subsequent retrograde urethrogram confirmed satisfactory healing. The patient experienced an adequate recovery and healing, and he was followed up for four months without any urinary symptoms. Isolated urethral injuries without an associated penile fracture are uncommon clinical entities. However, when they do occur, they carry a significant risk of complications if not promptly identified. Among the potential sequelae of delayed diagnosis are peri-urethral and scrotal abscesses, which can lead to increased morbidity. These injuries may present atypically, especially following coital trauma, making a high index of clinical suspicion important. Early recognition, supported by appropriate imaging and timely intervention, is key to preventing functional complications. This case highlights the importance of early diagnosis and prompt treatment in optimising patient outcomes and minimising the risk of long-term complications.

## Introduction

A penile fracture is a rupture involving the tunica albuginea of one or both corpora cavernosa. Concomitant urethral rupture is not common and occurs in 10% to 20% of penile fractures [1]. Isolated urethral injury without penile fracture from sexual intercourse is infrequent [2,3]. Penile fracture most commonly affects men between 30 and 50 years old, with a reported overall incidence in the United States of about 1 in 175,000 men [4].

Trauma during sexual relations is responsible for approximately one-third of all cases; the female-dominant position is most commonly reported [4]. Common causes of penile fracture include intercourse, masturbation, rolling over in bed, and forced flexion to achieve detumescence [4]. The most common cause of penile fracture is often during vigorous activity, with the “woman-on-top” position being identified as a precarious position [4]. Penile fracture most commonly occurs as a result of direct impact during sexual contact, leading to a sudden increase in pressure within the cavernosal tissue and subsequent rupture of the tunica albuginea [5]. Other mechanisms of injury include forceful bending of an erect penis, vigorous masturbation, and accidental trauma [5].

Penile fracture is characterised by a sudden cracking or popping sound, pain, and immediate detumescence [6]. Local swelling and discolouration of the penile shaft occur and may extend to the lower abdominal wall. On physical examination, the normal external penile appearance is totally obliterated due to significant penile deformity, swelling, and ecchymosis [4]. Diagnosis is made based on history and physical examination findings. Blood at the urethral meatus, hematuria, and difficulty voiding should raise the suspicion of a urethral injury [7]. Conditions that mimic penile fracture include penile injuries such as dorsal vein or artery rupture [8], penile contusion, paraphimosis, penile cellulitis, priapism, urethral tear, dependent oedema, and acute painless scrotal swelling [9].

Imaging modalities to confirm the diagnosis or exclude other concomitant injuries include ultrasonography, CT scan, retrograde urethrogram, and MRI [4,10]. Intraoperative flexible cystoscopy is often performed routinely to confirm or rule out urethral injury and to facilitate a safer catheter placement at the time of penile exploration [7]. Penile fracture is a urologic emergency; immediate surgical exploration and repair are recommended [4]. A neglected penile fracture could result in complications, including erectile dysfunction, abscess, and penile curvature [11].

While penile fractures with urethral involvement are more common, spongiosal injuries from coital trauma without cavernosal injuries, though rare, require prompt diagnosis and management to optimise outcomes and minimise complications. Although urethral injuries are not life-threatening, if not diagnosed early, they can cause morbidities, including peri-urethral abscess, scrotal abscess, and urethral stricture, among others. A high index of suspicion is essential in patients presenting with painful scrotal swelling mimicking epididymoorchitis. We report a case of isolated bulbar urethral injury following coital trauma.

## Case presentation

An 18-year-old male presented with two days of increasing penile and painful scrotal swelling following sexual intercourse, with the man-on-top position. He noticed minimal bleeding at the frenular region. He did not report visible hematuria, snapping or popping sound, nor immediate detumescence. There was no difficulty with voiding, and he achieved spontaneous erection thereafter. Significant examination findings included penile oedema, with minimal deformity and deviation to the left, intact prepuce, small laceration at the frenular region, diffuse scrotal swelling, erythematous and tender, with differential warmth. It was difficult to palpate the testes due to the oedema and tenderness.

**Figure 1:**
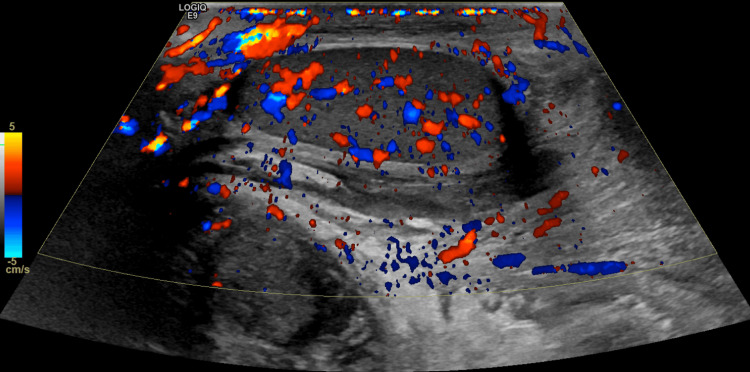
Ultrasonography of the left testis showing hypervascularity, suggesting severe epididymoorchitis.

Ultrasound of the testes, groin, and inguinal region on admission showed significant scrotal oedema with fat stranding/cobblestone appearances, which extended to the groin and lower abdomen. Hypervascularity was noted throughout both hemiscrotum, suggesting severe epididymoorchitis, which was worse on the right side (Figure 1). A diagnosis of epididymoorchitis was made based on clinical and radiological features. The C-reactive protein (CRP) and white blood cell (WBC) counts were elevated, with values of 189 mg/L and 15.31 × 10^9^/L, respectively. Urine dip and microscopy revealed no microscopic signs of a urinary tract infection. He started using antibiotics according to the Trust protocol. On admission, he had temperature spikes of 38.3-38.6°C. Blood culture grew methicillin-resistant *Staphylococcus aureus*. The antibiotics were changed based on the culture result.

**Figure 2:**
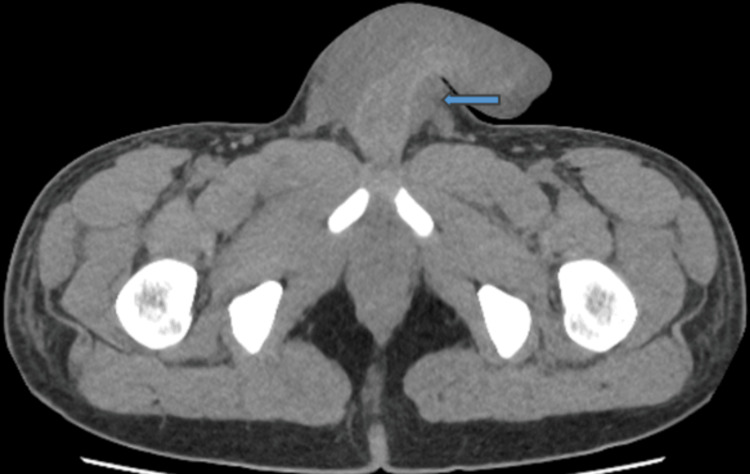
CT scan of the abdomen and pelvis: enhancing peri-urethral fluid collection, indicative of a large peri-urethral abscess.

**Figure 3:**
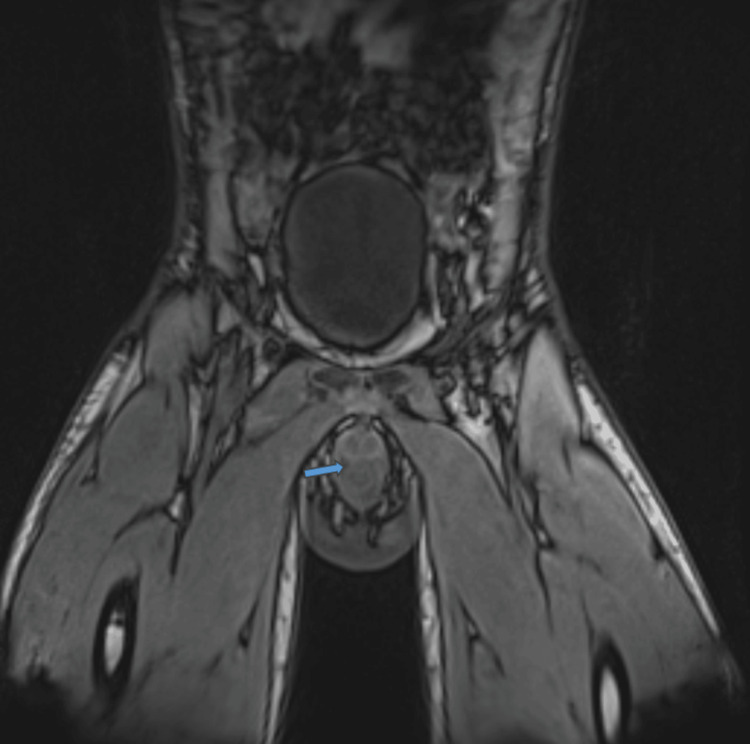
Pelvic MRI: a small tear involving the right lateral aspect of the bulbar urethra, measuring approximately 2.5 mm, along with a peri-urethral collection.

By the third day of admission, there was progressive improvement in the oedema and pain. However, WBC and CRP remained elevated, while the fever persisted. He was noted to have blood-stained discharge per urethra, which raised the suspicion of urethral injury. A CT scan of the abdomen and pelvis showed avid irregular and discontinuous enhancement of the penile urethra, suggesting a breach in the urethral wall, with a large, irregular, rim-enhancing fluid collection extending along the penile shaft, indicative of an extensive peri-urethral penile abscess. A 1 cm depth fluid rim, enhancing fluid collection around the right testicle, indicative of a scrotal abscess, appeared contiguous with this. Proximal extension of the abscess suggested to the proximal bulbar urethra, along with generalised penile and scrotal oedema (Figure 2). Pelvic MRI, post-contrast coronal sequences, indicated a small tear involving the right lateral aspect of the bulbar urethra. This tear measured approximately 2.5 mm. A peri-urethral collection was present, which appeared to be improving compared to the recent CT scan. The corpora cavernosa appeared unremarkable (Figure 3). Following these findings, a retrograde urethrogram (RUG) was not deemed necessary to further confirm urethral injury.

Under general anaesthesia, the patient underwent ultrasound-guided suprapubic cystostomy (SPC) using the Seldinger technique, followed by flexible cystoscopy and urethral catheterisation using a 16-Fr silicone Foley catheter over a guidewire, and scrotal exploration and drainage of a scrotal abscess. The intraoperative findings included bulbar urethral injury, normal bladder neck and bladder, and minimal pus tracking from the penis into the scrotum. The scrotal exploration was performed through a transverse hemiscrotal skin incision, with minimal tracking of pus from the penis. Copious warm saline lavage was done. A corrugated rubber drain was inserted. He was placed and maintained on continuous bladder drainage via both the SPC and urethral catheter.

Within 24 hours post-operation, the fever resolved. Two days later, a second look (wound inspection and change of wound dressing) was undertaken under general anaesthesia. Following the subsequent changes, wound dressings were initially applied daily and, later on, alternate days, at the bedside. The patient continued on oral antibiotics based on the sensitivity results.

**Figure 4:**
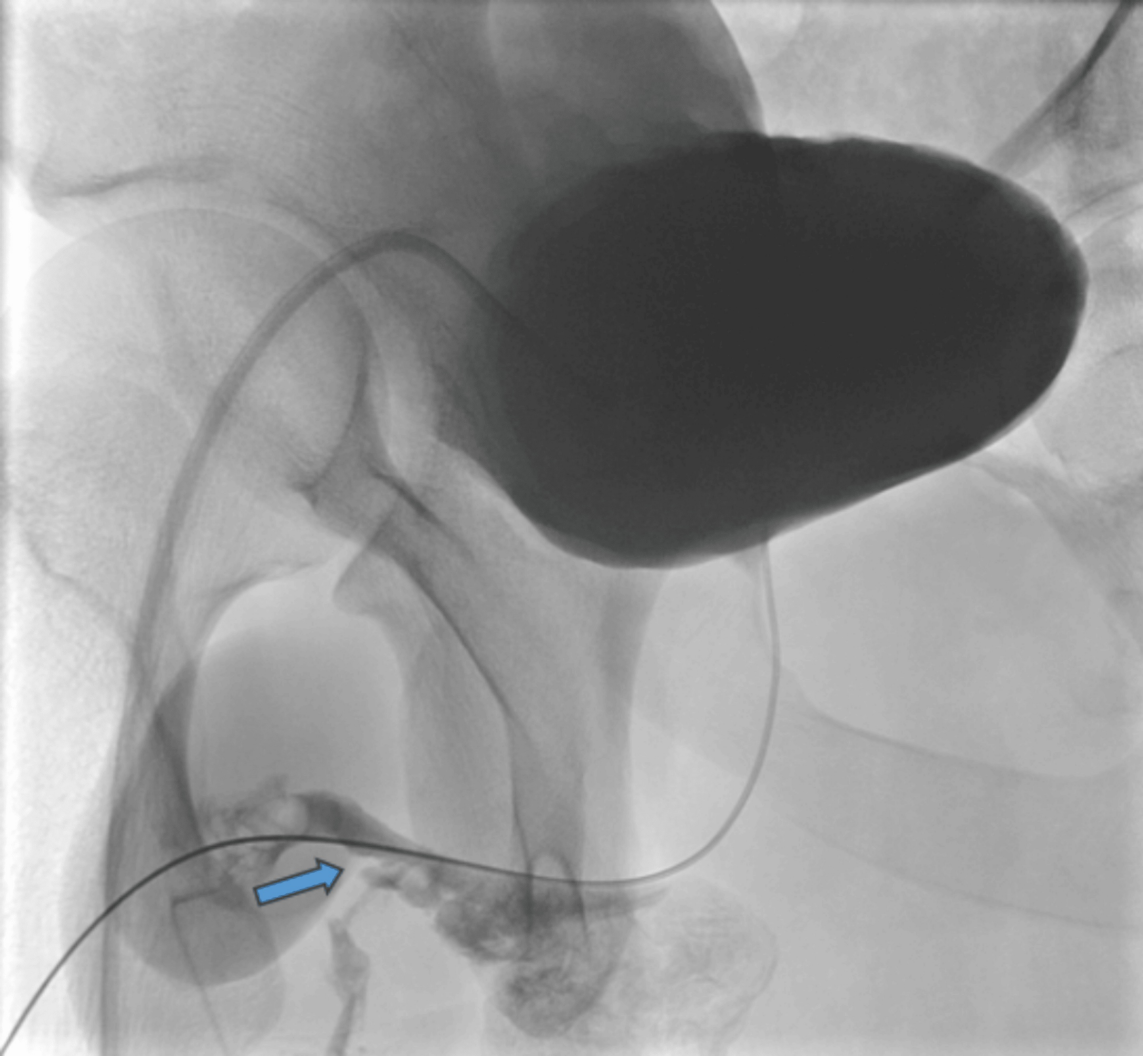
Contrast extravasation suggestive of incomplete healing on 20th day post-injury.

**Figure 5:**
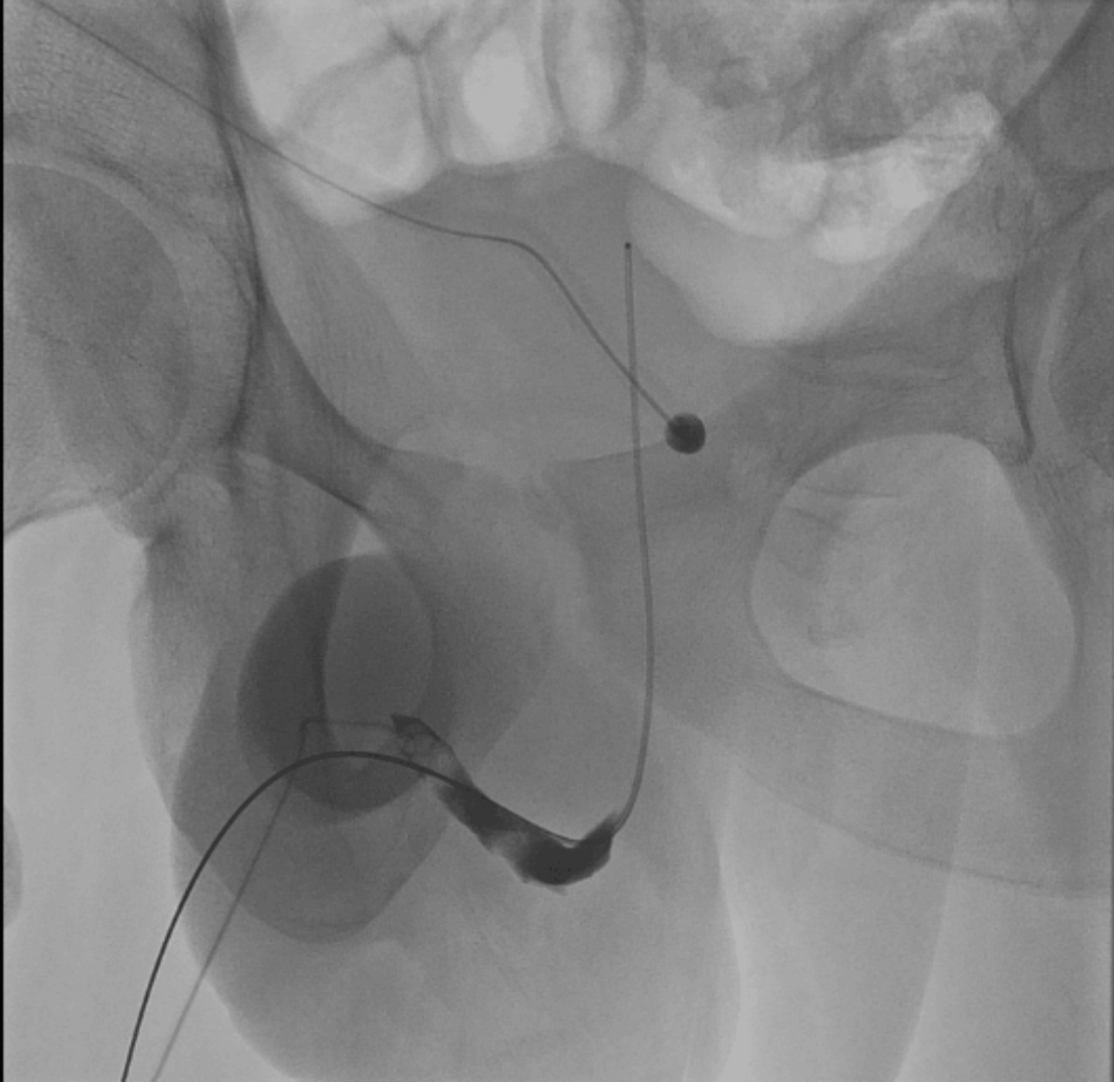
Retrograde urethrogram on the 30th day post-injury showing no contrast extravasation.

**Figure 6:**
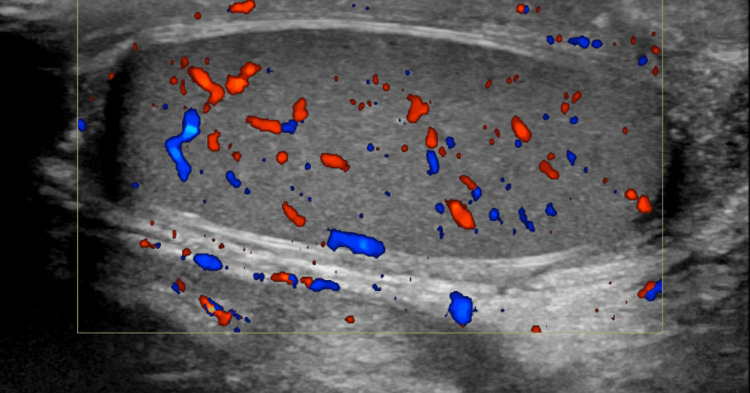
Ultrasonography of the right testes seven weeks post-injury showing normal findings with resolution of inflammation.

On the 20th day post-injury, he underwent an RUG, which showed minimal contrast extravasation, suggestive of incomplete healing (Figure 4). He was left with continuous bladder drainage via the SPC and a urethral catheter. Ten days after, a repeat RUG showed no extravasation with complete healing (Figure 5). The suprapubic catheter was spigoted while the patient voided per urethral, and was successfully removed two weeks after. A follow-up ultrasonography of the testes seven weeks post-injury showed no hyperaemia to suggest inflammatory changes, and no discrete collection was noted. Both epididymis and testes appear normal. No focal testicular abnormality was seen. No hydrocele or varicocele was noted (Figure 6). He was followed up for four months, with no change in erections and no urinary symptoms.

## Discussion

Penile fractures are uncommon urological emergencies [12]. Penile fracture with associated urethral rupture, which could be partial or complete, is a rare condition, with an occurrence varying from 1% to 38% [13]. Isolated corpus spongiosum and urethral injuries after male coital trauma are extremely rare [1]. Urethral injuries can first be classified based on location as either anterior or posterior [14]. Anterior urethral injuries, involving the penile and/or bulbar urethra, are often the result of blunt or penetrating trauma, with the bulbar urethra being the most common area affected [15]. The mechanisms underlying these injuries vary and may include iatrogenic causes or self-inflicted trauma resulting from sexual misadventures [15]. Additional etiologies encompass blunt force trauma to the perineum, such as those sustained from falls, straddle injuries, or sporting accidents. Notably, the majority of penile fractures (approximately 74.5%) occur during sexual intercourse, most frequently associated with the “doggy-style” and “man-on-top” positions [16]. Urethral involvement was observed in 10.6% of cases [16]. In rare cases, forceful or unusual sexual positions can lead to isolated urethral injury without a penile fracture [16]. Our patient had an isolated partial bulbar urethral injury occurring in the “man-on-top” position. Penile fracture routinely causes a cracking sound followed by rapid detumescence, sudden swelling, and ecchymosis of the penis, so that it achieves an appearance known as “eggplant deformity.” Our patient did not have these classical clinical presentation findings.

The cardinal presentation of urethral injury is blood at the meatus. Blood in the meatus, hematuria, and voiding symptoms are highly suggestive of urethral rupture, but the absence of these findings does not exclude urethral injuries [13]. Others include acute urinary retention, pain during urination, swelling, and bruising in the perineal area. In our patient, the bilateral tender scrotal swelling initially raised the suspicion of bilateral epididymoorchitis. However, the later finding of bloody discharge per urethra raised the suspicion of urethral injury, which was diagnosed by imaging (MRI preoperatively) and intraoperatively by flexible cystoscopy.

Urethral injury can result in a urine leak outside the damaged urethra into the surrounding tissues, causing inflammation, swelling, and an increased risk of infection. If left untreated, this can lead to the formation of peri-urethral abscesses and scrotal abscesses. Periurethral abscesses may exhibit a wide range of clinical features, including pain (scrotal, penile, pelvic, perineal, or suprapubic), fever, dysuria, pyuria, and acute urinary retention [17]. Open incision and drainage combined with antibiotics are the mainstay of management [17]. Our patient had peri-urethral and scrotal abscesses, which probably caused the persistent fever. These were sequelae of a delayed diagnosis due to an atypical presentation. Hence, a high index of suspicion is required in cases like this. Our patient underwent antibiotic treatment, incision and drainage, and SPC.

RUG is the diagnostic imaging study of choice in the setting of suspected urethral injury [15]. During RUG, any extravasation outside the urethra is pathognomonic for urethral injury. A small, flexible cystoscope can be inserted into the urethra to visualise the injury directly. Flexible urethrocystoscopy is a valuable alternative for diagnosing an acute urethral injury and can help differentiate between complete and partial ruptures. In our patient, flexible cystoscopy identified the site of urethral injury and was invaluable in the diagnosis. Depending on the suspected cause and location of the injury, imaging modalities such as CT scans or MRIs may be used as adjuncts to evaluation, especially to rule out cavernosal involvement in cases posing a diagnostic dilemma [12]. In our index case, both the CT scan and MRI served as diagnostic adjuncts, with the MRI detecting the site and extent, while also ruling out cavernosal injury.

The treatment of urethral injuries could be non-operative or operative, depending on the severity and location of the injury. Our patient had an incomplete bulbar urethral injury and was managed non-operatively with initial urinary diversion via the SPC and urethral catheterisation. Healing was confirmed with RUG showing no contrast extravasation. In some cases, endoscopic realignment, which involves guiding a catheter through the urethra to realign the injured area, is performed. In complete urethral discontinuity, surgery may be required after an initial urinary diversion via the SPC.

## Conclusions

Isolated urethral injuries without an associated penile fracture are uncommon clinical entities. However, when they do occur, they carry a significant risk of complications if not promptly identified. Among the potential sequelae of delayed diagnosis are peri-urethral and scrotal abscesses, which can lead to increased morbidity. These injuries may present atypically, especially following coital trauma, making a high index of clinical suspicion important. Early recognition, supported by appropriate imaging and timely intervention, is key to preventing functional complications. This case highlights the importance of early diagnosis and prompt treatment in optimising patient outcomes and minimising the risk of long-term complications.
